# Earthworms Drastically and Differentially Modify the Bacteriomes and Mycobiomes of Sewage Sludge

**DOI:** 10.3390/biotech15020033

**Published:** 2026-05-10

**Authors:** Marcos Pérez-Losada, Manuel Aira, Jorge Domínguez

**Affiliations:** 1 Computational Biology Institute, Department of Biostatistics and Bioinformatics, Milken Institute School of Public Health, George Washington University, Washington, DC 20052, USA; mlosada@gwu.edu; 2 Grupo de Ecoloxía Animal (GEA), Universidade de Vigo, 36310 Vigo, Spain; jdguez@uvigo.es

**Keywords:** 16S, bacteriome, ITS, earthworm, human pathogen, microbial communities, mycobiome, sewage sludge, vermicomposting

## Abstract

Sewage sludge management poses major environmental challenges due to increasing production and concerns about contaminants and microbial risks. Vermicomposting offers a sustainable biological treatment, yet the extent to which different earthworm species shape microbial outcomes remains poorly understood. Here, we examined how gut transit by three epigeic (*Eisenia andrei*, *E. fetida*, and *Dendrobaena hortensis*) and two anecic (*Lumbricus friendi* and *L. terrestris*) earthworm species alters bacterial and fungal communities in fresh sewage sludge. Using 16S rRNA and ITS amplicon sequencing combined with multivariate, differential-abundance, and functional prediction analyses, we compared sludge and earthworm cast bacteriomes and mycobiomes. Earthworm gut transit caused pronounced species-specific restructuring of bacterial and fungal community composition, diversity, and functional profiles, with clear separation between sludge and cast communities. Functional analyses indicated coordinated shifts in bacterial metabolic potential and fungal trophic modes consistent with enhanced biosynthetic and decomposer functions. Pathogen profiles were reshaped in a host-dependent manner, with low overall abundances and selective changes rather than uniform suppression. These findings demonstrate that vermicomposting outcomes depend strongly on earthworm species and microbial kingdom, highlighting the importance of earthworm lifestyle diversity when evaluating the ecological safety and agronomic potential of sludge-derived amendments.

## 1. Introduction

Sewage sludge is the semisolid organic by-product generated during municipal wastewater treatment and represents one of the most challenging waste streams to manage globally, as its production continues to rise and demands safe and sustainable disposal strategies [1,2,3,4]. Although sewage sludge has considerable potential as an organic soil amendment, its direct application is restricted by the presence of organic and inorganic contaminants [5]. In this context, biological stabilization technologies play a key role in circular waste management. While traditional composting provides partial microbial stabilization, vermicomposting has emerged as a highly effective alternative that converts sludge into high-quality biofertilizers and biostimulants [6,7,8].

Vermicomposting begins with the direct activity of earthworms. As ingested material passes through the earthworm gut, it undergoes substantial biochemical and microbiological modification, altering its physical and chemical properties. This transformation is not merely mechanical; it involves extensive restructuring of the original microbial communities and the introduction of gut-derived microorganisms [6,7,9]. Recent research demonstrates that vermicomposting profoundly reshapes both the taxonomic composition and functional potential of sewage sludge microbiomes, enriching bacterial groups associated with pathogen suppression and plant-growth promotion [10,11]. Furthermore, vermicomposting enhances microbial detoxification and substantially reduces biological hazards, thereby improving the safety of sludge for agricultural use [12,13].

However, an important nuance often overlooked in the literature is that the effects of vermicomposting are not uniform across earthworm species; the influence of earthworms on substrate microbiomes (e.g., microbial composition, structure, and diversity) is tightly linked to species-specific biology [9,14]. Each earthworm species produces castings with a distinct microbial signature, indicating that the biostimulant properties of the final vermicompost depend strongly on the species performing the transformation [9,14]. Despite this, research and industrial practice have traditionally focused on epigeic species such as *Eisenia andrei*, *E. fetida*, and *Dendrobaena hortensis*, whereas soil-burrowing anecic species such as *Lumbricus*, which dominate many sludge-amended agroecosystems, have been largely ignored [15]. Evaluating how different soil-engineer species process sewage sludge is therefore essential, as species-specific gut microbiomes and feeding strategies can drive markedly different patterns of contaminant transformation, pathogen reduction, and microbiome restructuring, ultimately determining the safety and agronomic value of the treated material [15].

In this study, we compared three epigeic vermicomposting species (*Eisenia fetida, E. andrei,* and *Dendrobaena hortensis*) with two anecic agricultural species (*Lumbricus friendi* and *L. terrestris*) in their capacity to process sewage sludge. Our primary objective was to assess how gut transit—the initial and critical phase of vermicomposting—modifies the composition and functional potential of bacterial and fungal communities present in fresh sludge. To address this, we combined next-generation sequencing of 16S rRNA and ITS regions with advanced bioinformatic and statistical analyses. We hypothesized that cast bacteriomes and mycobiomes exhibit species-specific taxonomic and functional signatures; therefore, gut transit in each earthworm species would restructure the sludge microbiome in distinct ways, ultimately shaping the properties and agronomic potential of the resulting organic amendment.

## 2. Materials and Methods

### 2.1. Sewage Sludge and Sampling of Earthworm Casts

Fresh sewage sludge was collected from a wastewater treatment plant located in Moaña (Galicia, Spain). Immediately after sampling, five aliquots of sludge (ss) were frozen at −80 °C, and the remaining material was used to establish the vermicomposting experiment. Fresh sludge was provided as feed to ongoing laboratory cultures of the earthworm species *Dendrobaena hortensis* (Dh), *Eisenia andrei* (Ea), *Eisenia fetida* (Ef), *Lumbricus friendi* (Lf), and *Lumbricus terrestris* (Lt) already feeding on sewage sludge. We selected the epigeic species Ea, Ef, and Dh because they are commonly used in research and industrial practices including vermicomposting; we also chose the anecic species Lr and Lt because they dominate many sludge-amended agroecosystems [16]. All earthworm cultures were maintained in the same conditions and processed identically. After 48 h of feeding, we collected 5–20 individuals per species (depending on the size of mature specimens) from each culture. A 48 h interval is considered sufficient for ingested material to pass through the earthworm gut and produce new casts [17,18,19]. Earthworms were sequentially washed with tap and sterile water, placed in sterile Petri dishes (five dishes per species) and incubated for 24 h to allow gut voiding [9]. Subsequently, freshly deposited cast samples were aseptically collected from each Petri dish (N = 5 per earthworm species) using a sterile spatula, and stored at −80 °C in Eppendorf tubes until further processing. This composite sampling procedure reduces within-sample variability and stabilizes species-level microbiome profiles.

### 2.2. Amplification, Sequencing and Analysis of 16S and ITS rRNA Genes

DNA was extracted from 5 fresh sewage sludge samples and 25 earthworm cast samples (0.25 g fresh weight) in a laminar flow hood to prevent contamination using the MO-BIO PowerSoil^®^ kit, Carlsbad, CA, USA, according to the manufacturer’s protocol. To characterize the bacteriome, we sequenced the V4 region of the 16S rRNA gene with primers 515F (GTGYCAGCMGCCGCGGTAA) and 806R (GGACTACNVGGGTWTCTAAT). To characterize the mycobiome, we sequenced the ITS rRNA gene with primers ITS1f (CTTGGTCATTTAGAGGAAGTAA) and ITS2 (GCTGCGTTCTTCATCGATGC). All libraries were run on a 2 × 250 Illumina MiSeq platform at Novogene, Cambridge, UK. All 16S and ITS sequence data have been deposited in the GenBank SRA database under accession numbers PRJNA1446263 and PRJNA1446293, for 16S and ITS rRNA, respectively.

### 2.3. Bioinformatic and Statistical Analysis

Amplicon sequence variants (ASVs) were inferred in DADA2 (version 1.34.0). We used ASVs instead of operational taxonomic units because they provide more accurate and reproducible results [20,21]. Raw sequences were processed using the DADA2 pipelines for 16S (https://benjjneb.github.io/dada2/tutorial.html accessed on 11 April 2025) and ITS (https://benjjneb.github.io/dada2/ITS_workflow.html accessed on 11 April 2025). 16S and ITS forward/reverse read pairs were filtered using standard filtering parameters: maxN = 0, truncQ = 2, rm.phix = TRUE and maxEE = 2, and truncLen = c(200,200). ASVs were independently inferred from the forward and reverse reads of each sample using the run-specific error rates, and the read pairs were then merged. Chimeras were identified in each sample and removed. The taxonomy of 16S and ITS ASVs was inferred using the RDP naive Bayesian classifier v2.14 [22] against the SILVA database (version 138.2) [23] and the UNITE database (version 10) [24], respectively, with a bootstrap confidence level of 80. ASVs that remained unclassified at the phylum level were excluded from further analysis.

Differences in bacterial and fungal abundances at the phylum and genus levels between sewage sludge and earthworm species were estimated using negative binomial models in DESeq2 v1.52.0 [25]. The Wald test was used to determine differential abundances of bacterial and fungal phyla and genera.

Microbial richness, evenness and relatedness (ASV Richness, Shannon, inverse Simpson and Phylogenetic Diversity indices) within samples (alpha-diversity) were estimated at the ASV level after rarefying to the minimum sample size. Significant differences in alpha-diversity estimates were assessed using the Kruskal–Wallis test followed by paired Wilcoxon tests. Taxonomic (Bray–Curtis and Jaccard) and phylogenetic (UNIFRAC) beta-diversity indices were also estimated at the ASV level using variance-stabilized transformation. Differences in beta-diversity between groups were assessed using permutational multivariate analysis of variance (PERMANOVA). We also used principal coordinates analysis (PCoA) to depict changes in beta-diversity across samples.

We estimated the abundance of human bacterial pathogens (HBPs) in the sewage sludge and earthworm bacteriomes as outlined in [26]. We applied the assignSpecies function in DADA2 v1.36 to achieve unambiguous taxonomic identifications at the species level, as suggested for 16S amplicon data [27]. Then, we crossed the ASV species-level taxonomy with the comprehensive list of HBPs reported by Barlett et al. [28]. Similarly, we also conducted an analysis of human fungal pathogens (HFPs) in the mycobiomes as outlined in [29]. We created a list of fungal pathogens by gathering information from different sources. We used the R package and database FUNGuild v0.3.0, accessed in February 2025 [30], a tool that assigns trait information by mapping ASVs to a taxonomic classification. We only included ASVs rated as “probable” and “highly probable” in the analysis. We also included fungal pathogens in the EuPathDB database [31], the WHO report [32] (accessed on 12 January 2025), and the HFP in the One Health: Fungal Pathogens of Humans, Animals, and Plants report [33] (accessed on 12 January 2025). Lastly, we also included the fungal pathogenic genera identified by [34]. Our final list of HFP included 40 genera and 137 species. Differences in HBP and HFP abundance between sewage sludge and earthworm species were assessed using DESeq2 v1.52.0 as described above.

Bacterial functional profiles were predicted using the Phylogenetic Investigation of Communities by Reconstruction of Unobserved States (PICRUSt2) software package [35]—following the protocol of standard full pipeline (https://github.com/picrust/picrust2/wiki/Full-pipeline-script accessed on 12 November 2025) and KEGGREST v1.44.0 [36]. Beta-diversity analyses of functional profiles were carried out as indicated above for taxonomic distances and tested via PERMANOVA. As in [9,12,26], we compared metabolic pathways related to amino acid biosynthesis, furfural and bisphenol degradation, antibiotic synthesis, antibiotic resistance, plant hormone synthesis and nitrogen metabolism between sewage sludge and earthworm species. We focused on these functional categories because they capture key ecological processes that determine the biotransformation capacity, ecological safety, and agronomic potential of fresh and sludge-transformed microbiomes. Differences in functional category abundance were assessed using Kruskal–Wallis and Wilcoxon tests.

Fungal ecological trophic modes were compared between sewage sludge and earthworm mycobiomes using the FUNGuild database classification [30], accessed in February 2026. As for HFP, we only included in the analysis ASVs rated as “probable” and “highly probable”. Differences in trophic mode abundance were assessed using the Kruskal–Wallis test and the Wilcoxon test.

All the amplicon sequence data, bioinformatic and statistical analyses were carried out in R v4.6.0 and RStudio v2026.4.0.526 using the R packages phyloseq v1.56.0 [37], tidyverse v2.0.0 [38], vegan v2.7.3 [39], rstatix v0.7.3 [40], and microeco v2.1.0 [41]. Statistical approaches appropriate for small sample sizes were applied to limit false positive findings under the assumptions of the respective tests. Nevertheless, the limited number of independent samples in our study can reduce statistical power and may increase the likelihood of Type II error; therefore, non-significant results should be interpreted cautiously. All *p* values were corrected for multiple comparisons using the FDR method.

## 3. Results

We analyzed 5 samples of fresh sewage sludge (ss) and 25 of earthworm casts of the species *D. hortensis* (Dh), *E. andrei* (Ea), *E. fetida* (Ef), *L. friendi* (Lf), and *L. terrestris* (Lt). We sequenced the variable V4 region of the 16S rRNA gene (~250 bp) to characterize the bacteriome and the ITS gene (~250 bp) to characterize the mycobiome.

### 3.1. Taxonomic Diversity and Microbial Structure of the Microbiomes

The bacteriome of the 30 samples after quality control included 1,702,211 reads, ranging from 49,177 to 64,621 reads per sample (mean = 56,740.4), and 4296 ASVs. All samples showed adequate levels of diversity for microbiome analyses as indicated by rarefaction curves of ASV richness (Supplementary Materials Figure S1). The ss bacteriome included 923 unique ASVs, while the earthworm cast bacteriomes contained 178 (Ef) to 1030 ASVs (Lf) (Supplementary Materials Figure S2). The ss bacteriome shared a low number of ASVs with the earthworm casts, ranging from 1 (Dh and Lf) to 51 (Ea).

**Figure 1:**
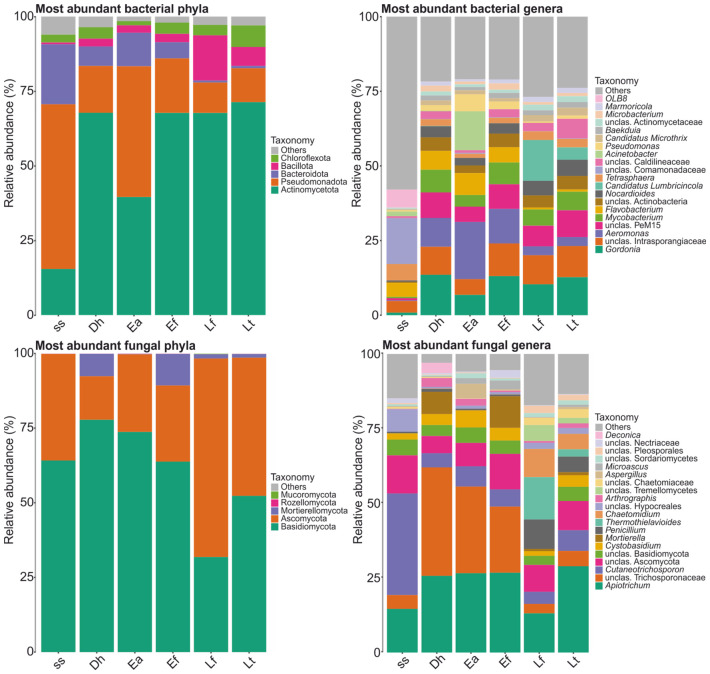
Relative mean abundance of most abundant phyla and genera in the bacteriomes and mycobiomes of sewage sludge (ss) and casts of earthworms *Dendrobaena hortensis* (Db), *Eisenia andrei* (Ea), *Eisenia fetida* (Ef), *Lumbricus friendi* (Lf), and *Lumbricus terrestris* (Lt).

The ss bacteriome was dominated by the phyla Pseudomonadota (55.1% average), Bacteroidota (20.1%), and Actinomycetota (15.5%); while the earthworm cast bacteriomes were dominated by Actinomycetota (39.6–71.3% average) and Pseudomonadota (10.2–43.8%), and variable proportions of Bacteroidota (0.7–11.2%) and Bacillota (2.5–15.2%) (Figure 1 and Supplementary Materials Table S1). At the genus level, the ss bacteriome mainly comprised unclassified Comamonadaceae (15.3% average), *OLB8* (5.9%), and *Tetrasphaera* (5.5%); while the earthworm cast bacteriomes mainly comprised *Gordonia* (6.8–13.6% average), unclassified Intrasporangiaceae (5.3–10.9%), *Aeromonas* (2.9–19.1%), unclassified PeM15 (5.1–9.0%), and *Mycobacterium* (3.9–7.6%) (Figure 1 and Supplementary Materials Table S1).

Up to 18 phyla showed significant Padj ≤ 0.027) differences in abundances between ss and the earthworm casts, with Dh and Lt showing the highest number (16 phyla) and Ef and Lf showing the lowest (14 phyla) (Supplementary Materials Table S2). Up to 273 genera showed significant (Padj ≤ 0.025) differences in abundance between ss and the earthworm casts, with Lt showing the highest number (219 genera) and Ea showing the lowest (179 genera) (Supplementary Materials Table S2).

**Figure 2:**
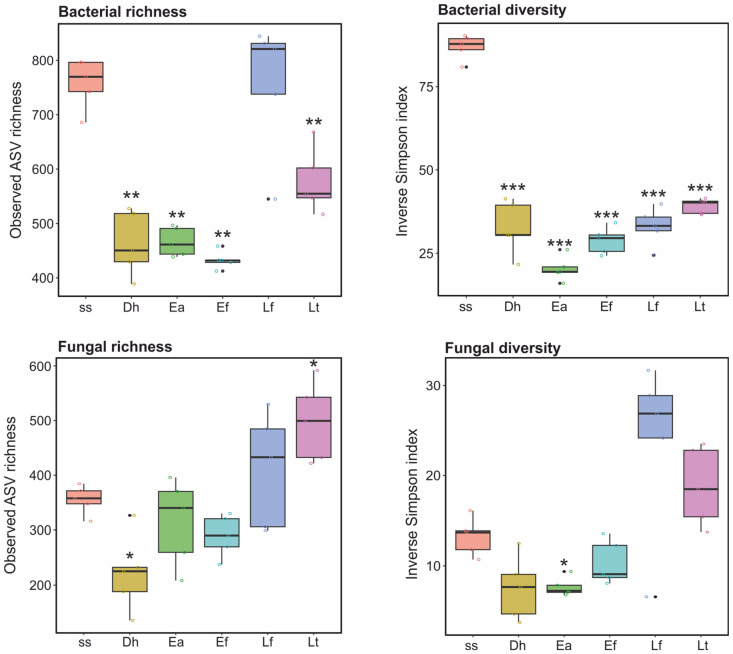
Alpha-diversity estimates in bacteriomes and mycobiomes of sewage sludge (ss) and casts of earthworms *Dendrobaena hortensis* (Db), *Eisenia andrei* (Ea), *Eisenia fetida* (Ef), *Lumbricus friendi* (Lf), and *Lumbricus terrestris* (Lt). Asterisks denote statistically significant pairwise comparisons between ss and earthworm casts (* = Padj ≤ 0.05, ** = Padj ≤ 0.01, *** = Padj ≤ 0.001).

Alpha-diversity estimates of richness, evenness and relatedness (ASV Richness, Shannon, Inverse Simpson, and phylogenetic diversity) decreased between ss and the earthworm casts for all indices except ASV Richness for Lf (Figure 2, Supplementary Materials Figure S3, and Supplementary Materials Table S3). All comparisons between ss and the earthworm casts (except ASV Richness for ss–Lf) resulted in significantly different (Padj ≤ 0.016) (Supplementary Materials Table S4).

**Figure 3:**
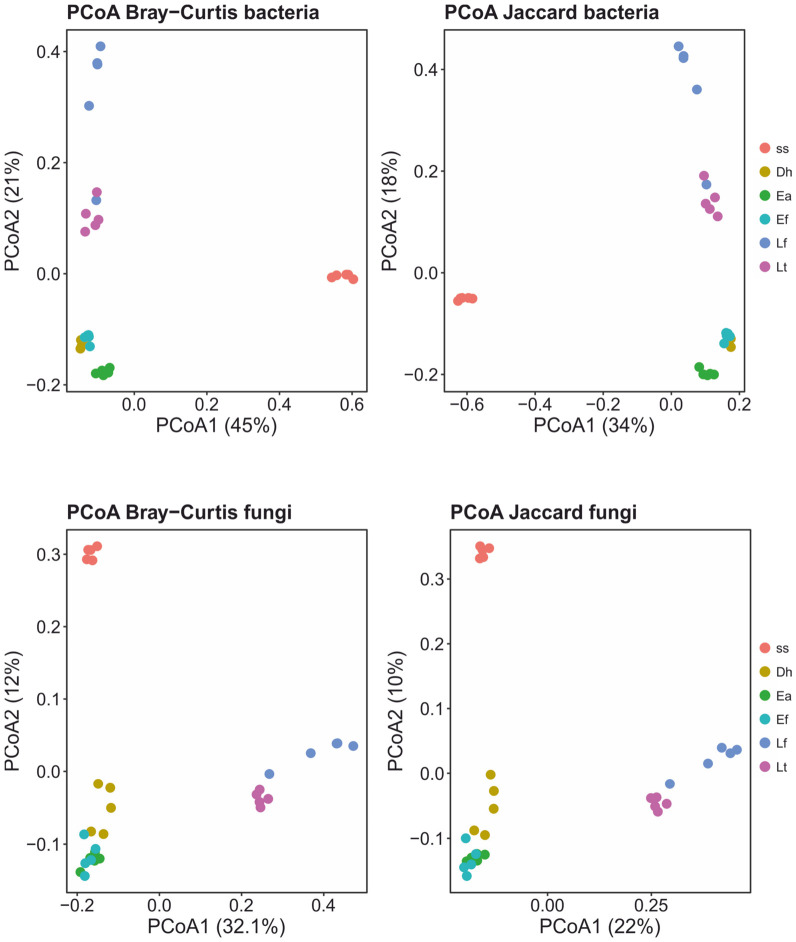
PCoAs of compositional beta-diversity estimates in bacteriomes and mycobiomes of sewage sludge (ss) and casts of earthworms *Dendrobaena hortensis* (Db), *Eisenia andrei* (Ea), *Eisenia fetida* (Ef), *Lumbricus friendi* (Lf), and *Lumbricus terrestris* (Lt).

Our PCoAs (Figure 3 and Supplementary Materials Figure S4) of beta-diversity estimates showed a clear segregation of the ss samples from the earthworm cast samples across all estimated distances (Unifrac, Bray–Curtis and Jaccard). The PERMANOVA analyses detected significant differences (Padj ≤ 0.016) in community structure (beta-diversity) between ss and all the earthworm casts (Supplementary Materials Table S5). The variance explained by the R^2^ statistic ranged from 53.7 to 96.8%.

We detected 29 human bacterial pathogenic species (HBP) in the bacteriomes of all our samples, ranging from 8 in Ea to 11 in ss and Dh (Supplementary Materials Table S6). Their mean microbial abundances were very low, ranging from 0.08 mean reads (*Bacteroides stercoris*) to 97.1 mean reads (*Turicibacter sanguinis*) and varied across groups, with ss showing the lowest means for all pathogens. Only five HBP (*Turicibacter sanguinis, Schaalia odontolytica, Sarcina ventriculi, Empedobacter falsenii,* and *Schaalia odontolyticass*) showed a significant (Padj < 0.05) increase in abundance in one to three of the earthworm casts compared to ss.

The mycobiome comprised 2,158,265 reads, ranging from 10,709 to 184,777 reads per sample (mean = 71,942.1), and 4063 fungal ASVs. All samples showed adequate levels of diversity for microbiome analyses as indicated by rarefaction curves of ASV Richness (Supplementary Materials Figure S1). The ss mycobiome included 479 unique ASVs, while the earthworm cast mycobiomes contained 161 ASVs (Dh) to 352 (Lt) (Supplementary Materials Figure S2). The ss mycobiome shared a low number of ASVs with the earthworm casts, ranging from 3 (Dh and Lt) to 35 (Ea).

Both ss and earthworm cast mycobiomes were dominated by the same two phyla Ascomycota (26.1 to 67.5% average) and Basidiomycota (30.8 and 74.2% average), with Mortierellomycota showing a ~10% relative mean abundance in Dh and Ef (Figure 1 and Supplementary Materials Table S1). At the genus level, the ss mycobiome mainly comprised *Cutaneotrichosporon* (34.0% average), *Apiotrichum* (14.6%), unclassified Hypocreales (7.6%), and unclassified Basidiomycota (5.3%); while the earthworm cast mycobiomes mainly comprised unclassified Trichosporonaceae (2.9–32.5% average), *Apiotrichum* (12.6–28.3%), and unclassified Ascomycota (6.5–11.8%) (Figure 1 and Supplementary Materials Table S1).

Up to 5 phyla showed significant (Padj ≤ 0.009) differences in abundance between ss and the earthworm casts for 1 to 4 of those phyla (Supplementary Materials Table S2). Up to 73 genera showed significant (Padj ≤ 0.003) differences in abundance between ss and the earthworm casts, with Lf showing the highest number of genera (63) and Dh and Ea showing the lowest (25) (Supplementary Materials Table S2).

Alpha-diversity estimates of richness and evenness (ASV Richness, Shannon, and Inverse Simpson) decreased in Dh, Ea and Ef casts, and increased in Lf and Lt casts compared to ss (Figure 2, Supplementary Materials Figure S3, and Supplementary Materials Table S3). Comparisons between ss and the earthworm casts resulted in significant (Padj ≤ 0.04) differences for one or two earthworm species casts across the three indices (Supplementary Materials Table S4).

The PCoAs (Figure 3) of beta-diversity estimates showed a clear segregation of the ss samples from the earthworm cast samples for both estimated distances (Bray–Curtis and Jaccard). The PERMANOVA analyses detected significant differences (Padj ≤ 0.015) in community structure (beta-diversity) between ss and the earthworm casts (Supplementary Materials Table S5). The variance explained by the R^2^ statistic ranged from 18.9 to 59%.

We detected 76 human fungal pathogenic (HFP) species in the mycobiomes of all our samples, ranging from 35 in Dh to 49 in ss and Lt casts (Supplementary Materials Table S6). Their mean microbial abundances ranged from 0.07 mean reads (*Circinella umbellata*, *Gongronella butleri* and *Penicillium tealii*) to 2518 mean reads (*Cystobasidium slooffiae*) and varied inconsistently (up and down) across groups, ranging from four HFP (Padj < 0.05) in ss vs. Dh casts to nine in ss vs. Lt casts.

### 3.2. Functional Diversity of the Microbiomes

**Figure 4:**
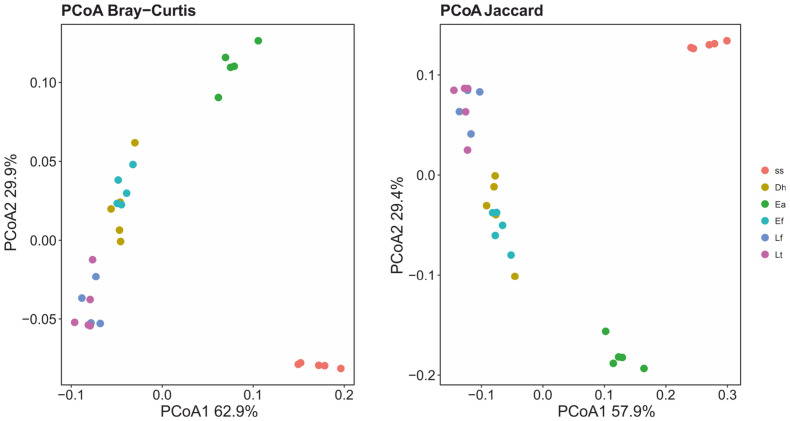
PCoAs of functional beta-diversity estimates in bacteriomes and mycobiomes of sewage sludge (ss) and casts of earthworms *Dendrobaena hortensis* (Db), *Eisenia andrei* (Ea), *Eisenia fetida* (Ef), *Lumbricus friendi* (Lf), and *Lumbricus terrestris* (Lt).

The PCoAs (Figure 4) of predicted functional properties showed a clear segregation of the ss samples from the earthworm cast samples for the estimated distances (Bray–Curtis and Jaccard). The PERMANOVA analyses detected significant differences (Padj ≤ 0.013) in functional structure between ss and the earthworm casts (Supplementary Materials Table S7). The variance explained by the R^2^ statistic ranged from 78.1 to 97.3%.

We compared the functional potential (PICRUSt2) of the ss and earthworm cast bacteriomes across several selected metabolic categories (Supplementary Materials Figure S5). The abundance of genes (significant pairwise comparisons in parenthesis) associated with antibiotic resistance (all significant; Padj ≤ 0.018), antibiotic synthesis (Lf and Lt significant; Padj < 0.001), biosynthesis of amino acids (all but Lf significant; Padj ≤ 0.021), and plant hormone synthesis (all but Ea significant; Padj ≤ 0.016) increased in all earthworm casts (Supplementary Table S8). Bisphenol degradation (all non-significant) and furfural degradation (Dh, Ea, and Lf significant; Padj < 0.049) decreased. Nitrogen metabolism (all non-significant) remained relatively stable across all bacteriomes (Supplementary Materials Table S8).

We compared seven fungal trophic modes (FUNGuild) between ss and the earthworm cast mycobiomes (Supplementary Materials Figure S6). Fungal richness (significant pairwise comparisons in parentheses) increased for all pairwise comparisons of Pathotrophs (Lt; Padj < 0.001), Saprotrophs (all significant but Ef; Padj ≤ 0.015), Saprotroph−Symbiotroph (Lt; Padj = 0.013), and Pathotroph−Saprotroph−Symbiotroph (all significant but Dh; Padj ≤ 0.013); while varied up and down by earthworm species in Pathotroph−Saprotroph (Dh and Lt; Padj < 0.001) and Pathotroph−Symbiotroph (Lt; Padj = 0.013) (Supplementary Materials Table S9).

## 4. Discussion

This study addresses how gut transit in three epigeic vermicomposting species (*Eisenia fetida, E. andrei,* and *Dendrobaena hortensis*) and two anecic agricultural species (*Lumbricus friendi* and *L. terrestris*) modifies taxonomic and functional profiles of bacteriomes (via 16S) and mycobiomes (via ITS) in fresh sewage sludge.

### 4.1. Earthworm Gut Transit Generates Taxonomic Restructuring of Sludge Microbiomes

Our results show that earthworm gut transit caused a pronounced and species-specific restructuring of both bacterial and fungal community composition relative to fresh sewage sludge (Figure 1, Supplementary Materials Figure S2, and Supplementary Materials Tables S1 and S2). For bacteria, this was reflected in a strong reduction in shared ASVs between sludge and casts, major significant shifts in phylum-level dominance from Pseudomonadota–Bacteroidota-rich sludge communities to Actinomycetota-dominated cast communities, and extensive significant genus-level turnover across earthworm species. These compositional shifts indicate that earthworm gut transit functions as a strong ecological filter, selectively eliminating most sludge-associated microorganisms while promoting taxa adapted to gut-associated and post-digestive environments [10,12,26]. The sharp reduction in shared ASVs shows that earthworm digestion imposes intense selective pressures through enzymatic activity, antimicrobial compounds, and fluctuating redox conditions [9]. The consistent enrichment of Actinomycetota in casts is particularly relevant, as this group is widely associated with organic matter decomposition, secondary metabolite production, and disease-suppressive soils, helping explain the beneficial properties often attributed to vermicompost [10,42,43,44,45]. Importantly, the magnitude of taxonomic restructuring differed among earthworm species, reinforcing earlier findings that host taxonomy is a primary determinant of cast microbiome composition under homogeneous feeding conditions [9,10,14,26].

Fungal communities also showed a strong reduction in shared ASVs, retained dominance of Ascomycota and Basidiomycota across all samples, but exhibited significant species-dependent changes in phylum proportions and extensive genus-level reassembly (Figure 1, Supplementary Materials Figure S2, and Supplementary Materials Tables S1 and S2). The persistence of dominant phyla alongside strong genus-level turnover suggests that gut transit reorganizes fungal communities rather than completely replacing them, consistent with previous observations of selective fungal filtering during vermicomposting [9,26,29]. This also suggests that fungal communities may undergo reorganization primarily through post-egestion regrowth and competitive dynamics in casts.

Differences between bacterial and fungal responses to earthworm gut transit likely reflect fundamental contrasts in their physiology, life-history strategies, and structural resilience. Bacteria are generally unicellular, fast-growing, and highly sensitive to rapid changes in redox conditions, pH, digestive enzymes, and antimicrobial compounds encountered in the earthworm gut, leading to strong taxonomic turnover and selective enrichment of gut-adapted taxa [46,47]. In contrast, many fungi possess multicellular hyphal networks, melanized cell walls, and resistant resting structures such as spores or sclerotia, which can survive gut passage more readily and subsequently regenerate in casts [46,47]. This structural resilience likely explains the persistence of dominant fungal phyla despite extensive genus-level reassembly. Additionally, fungi often occupy broader functional niches related to substrate decomposition and may be less tightly coupled to short gut residence times than bacteria, which respond more rapidly to selective pressures during digestion.

Alpha-diversity analyses revealed contrasting bacterial and fungal responses to earthworm gut transit (Figure 2; Supplementary Materials Figure S3, Supplementary Materials Tables S3 and S4). Bacterial richness, evenness, and phylogenetic diversity consistently declined in earthworm casts relative to sewage sludge across all earthworm species, whereas fungal diversity responses were more variable: fungal richness and evenness decreased in epigeic species but increased in anecic species. The consistent reduction in bacterial alpha diversity supports the view that earthworm digestion acts as a strong bottleneck, simplifying bacterial communities by selectively favoring taxa capable of surviving gut passage [9]. In contrast, the divergent patterns of fungal diversity suggest that fungi respond to gut transit through mechanisms distinct from those of bacteria [9,29]. Increased fungal diversity in soil anecic species may reflect broader feeding strategies and greater incorporation of soil-associated fungi, whereas epigeic species, which primarily process surface organic matter, may exert stronger filtering effects. These findings align with growing evidence that fungal communities are shaped not only by gut conditions but also by earthworm ecological strategies and habitat use [14,26,29,34].

Beta-diversity analyses demonstrated a clear and consistent separation between sewage sludge and earthworm casts for both bacterial and fungal communities across all distance metrics tested (Figure 3 and Supplementary Materials Figure S4). PERMANOVA results confirmed significant differences in community structure between sludge and each earthworm species casts, with high R^2^ values indicating that gut transit explains a substantial proportion of total community variation (Supplementary Materials Table S5). These patterns indicate that vermicomposting rapidly drives microbial communities toward distinct, earthworm-associated states, confirming that gut transit represents the primary phase of microbiome reassembly [6,7,48]. The strong beta-diversity separation observed for both bacteria and fungi reinforces the conclusion that vermicomposting is not merely a gradual modification of sludge microbiomes, but rather a rapid ecological transition driven by host-specific selection. The species-dependent clustering further emphasizes that different earthworm species generate distinct microbial trajectories, an aspect often overlooked in vermicomposting studies that focus on a single epigeic species [9,10,14].

### 4.2. Earthworm Gut Transit Reshapes Pathogen Load in Sewage Sludge

We detected 29 human bacterial pathogenic (HBP) species across all samples with very low overall abundances (0.1 to 97 mean reads) (Supplementary Materials Table S6). Notably, only five HBP were significantly enriched in earthworm casts relative to sludge. In contrast, human fungal pathogens (HFP) were more diverse (76 species detected; 0.1 to 2518 mean reads) and exhibited variable rates of enrichment and depletion across earthworms (particularly in anecic taxa) relative to ss (Supplementary Materials Table S6). These results suggest that earthworm gut transit does not uniformly suppress all pathogenic taxa, and its effect may vary across microbial kingdoms [26,29].

For HBP, earthworm digestion may act primarily as a selective ecological filter rather than a uniform inactivation process. Gut transit eliminates many dominant sludge-associated bacteria, altering competitive interactions and resource availability in the post-digestive environment. This restructuring can lead to competitive release, allowing a small subset of stress-tolerant or fast-growing opportunistic bacteria to increase in relative abundance without representing a true pathogen outbreak. Similar patterns have been reported in previous vermicomposting studies, which showed that increases in specific HBP are typically low in absolute abundance, transient, and dependent on earthworm species, sewage sludge, and gut conditions rather than indicative of increased health risk [26,29,34,49,50,51].

Variation in HFP rates and persistence after vermicomposting reflect biological traits fundamentally different from those of bacteria. Many fungal pathogens possess spores, thick cell walls, and high stress tolerance, enabling them to survive digestive processes and fluctuate in abundance as microbial communities reorganize [32,33,50]. Recent surveys of sludge- and wastewater-associated fungi emphasize that biological treatment processes frequently reshape fungal pathogen assemblages through community reassembly rather than elimination, a pattern consistent with the resilience of fungi to environmental stressors [26,29,34]. Earthworm species seem to further modulate these outcomes, as differences in feeding strategies and habitat use influence which fungal taxa are retained or redistributed following gut passage.

### 4.3. Earthworm Gut Transit Generates Functional Restructuring of Sludge Microbiomes

PCoA ordinations of predicted bacterial functional profiles (PICRUSt2) showed a clear separation between sewage sludge and earthworm casts across all distance metrics (Figure 4), with PERMANOVA indicating significant differences and high proportions of explained variance among earthworm species (Supplementary Materials Table S7). This suggests that bacterial taxonomic restructuring during gut transit is accompanied by a coordinated reorganization of metabolic potential, rather than functional redundancy masking compositional change. This tight coupling between taxonomic and functional turnover has been reported previously during vermicomposting and supports the view that earthworm gut transit selects for functionally coherent microbial assemblages adapted to post-digestive environments [9,10,52,53].

Comparisons of predicted bacterial functional categories revealed consistent shifts between sewage sludge and earthworm casts across all species (Supplementary Materials Figure S5 and Supplementary Materials Table S8). Pathways related to amino acid biosynthesis, antibiotic synthesis, antibiotic resistance, and plant hormone synthesis were generally enriched in earthworm-processed sludge, whereas pathways associated with bisphenol and furfural degradation decreased, and nitrogen metabolism remained comparatively stable. The enrichment of biosynthetic and secondary-metabolite pathways is consistent with previous studies linking vermicomposting to microbial functions associated with nutrient turnover, microbial competition, and plant growth promotion [9,10]. Increased representation of antibiotic synthesis and resistance pathways likely reflects enrichment of taxa such as Actinomycetota and Proteobacteria, which commonly harbor these functions and contribute to microbial community stabilization rather than increased antimicrobial risk [10,42,45,54,55,56]. The relative stability of nitrogen metabolism suggests functional redundancy and conservation of core nutrient-cycling processes, supporting the agronomic reliability of vermicomposted products. In contrast, the reduced representation of certain xenobiotic-degradation pathways indicates that detoxification capacity may depend on specific microbial consortia present in fresh sludge and selectively retained during gut transit, reinforcing previous observations that vermicomposting effects on detoxification are pathway-specific rather than universal [9,10,12,49,57].

Analysis of fungal ecological guilds revealed significant shifts in trophic mode composition between sewage sludge and earthworm casts, with enrichment of saprotrophic and mixed trophic categories in several earthworm species and variable responses across epigeic and anecic taxa (Supplementary Materials Figure S6 and Supplementary Materials Table S9). These shifts suggest that vermicomposting promotes fungal functional strategies associated with organic matter decomposition and nutrient recycling, reflecting changes in substrate quality and microbial competition following gut transit. Previous studies have shown that earthworm activity alters fungal trophic structure by modifying habitat conditions, redistributing organic resources, and selectively favoring fungi adapted to decomposer roles [9,34]. The greater variability observed among anecic species is consistent with their broader feeding strategies and stronger interaction with soil-derived fungal reservoirs, which may facilitate incorporation and persistence of diverse trophic modes during sludge processing.

### 4.4. Implications for Vermicomposting Strategies

Together, our findings demonstrate that vermicomposting is not a uniform or interchangeable process, but rather a species-dependent ecological transformation with direct consequences for the biosafety, functional capacity, and agronomic value of sewage sludge–derived products. Earthworm gut transit reshapes microbial communities in ways that depend not only on the substrate but also on earthworm identity, lifestyle, and feeding ecology. While epigeic species such as *E. andrei* and *E. fetida* produced relatively consistent and pronounced microbial restructuring, anecic species exerted distinct and sometimes contrasting effects on bacterial and fungal assemblages, functional profiles, and pathogen dynamics. These differences suggest that earthworm species selection can influence both the predictability and the ecological outcomes of vermicomposting processes.

From an applied perspective, these results also have important implications for sludge management and the design of sustainable vermicomposting systems. Epigeic species, which are commonly used in engineered vermicomposting operations, remain well suited for controlled waste processing due to their rapid feeding rates, strong microbial filtering capacity, and reproducible effects on community composition. However, the distinct microbial signatures associated with anecic species —dominant in many agroecosystems and natural soils— highlight their potential role in shaping post-application microbial trajectories when vermicompost is introduced into field soils. The host-dependent reshaping of pathogen profiles observed here, characterized by selective changes rather than universal suppression, further underscores the need for species-specific risk assessments rather than assumptions of generalized pathogen reduction during vermicomposting. From a human-health perspective, this is particularly relevant because sewage sludge can act as a reservoir for opportunistic and clinically relevant microorganisms, and changes during vermicomposting may alter exposure risks rather than simply eliminate them. Earthworm-driven shifts in pathogen composition could influence the persistence, regrowth, or ecological competitiveness of specific taxa following land application, especially in agricultural settings where human contact pathways exist. Consequently, evaluating vermicomposting outcomes through a species-specific and pathogen-focused lens is essential for accurately assessing biosafety and ensuring that sludge-derived amendments do not pose unintended public-health risks.

Lastly, our results argue for moving beyond single-species paradigms toward vermicomposting strategies that explicitly consider earthworm lifestyle diversity and ecological context. Integrating multiple earthworm functional groups, or selecting species based on targeted outcomes such as enhanced decomposer activity, biosynthetic potential, or compatibility with recipient soils, may improve both the safety and agronomic performance of sludge-derived amendments. By linking taxonomic composition, functional potential, and trophic structure of microbial communities to earthworm identity, this study provides a framework for tailoring vermicomposting practices to specific management goals, land-use scenarios, and regulatory requirements, thereby supporting more effective and ecologically informed recycling of organic wastes.

This study has several limitations. Our experimental design is cross-sectional; therefore, causality cannot be inferred, and the results are primarily descriptive. The genomic data collected (16S rRNA and ITS amplicon sequencing) have limited taxonomic resolution, provide only predicted functional profiles, and do not allow for in-depth characterization of the biological mechanisms driving the observed differences. We collected five composite samples of casts from 5 to 20 specimens per earthworm species, depending on the size of mature individuals. This limited number of independent samples may reduce statistical power and increase the likelihood of Type II error; therefore, non-significant results should be interpreted cautiously. Moreover, some clustering patterns may appear to align with ecological or taxonomic groupings, but we caution against overinterpretation given the limited representation and lack of experimental design to formally test these effects. Further research using longitudinal and a more robust experimental design (including additional variables such as seasonality, sludge treatments and physicochemical properties, ecological type, and evolutionary relatedness), as well as shotgun metagenomics, would be needed to fully explore the complex interactions occurring within these microbial communities.

## Data Availability

Sequence files and associated metadata and BioSample attributes have been deposited in the NCBI (PRJNA1446263 and PRJNA1446293).

## Supplementary Materials

The following supporting information can be downloaded at: https://www.mdpi.com/article/10.3390/biotech15020033/s1, Figures: Supplementary Materials Figure S1: Rarefaction curves of ASV Richness of the bacteriomes and mycobiomes of sewage sludge (ss) and casts from earthworms *Dendrobaena hortensis* (Db), *Eisenia andrei* (Ea), *Eisenia fetida* (Ef), *Lumbricus friendi* (Lf), and *Lumbricus terrestris* (Lt). Supplementary Materials Figure S2: UpSet plots of ASVs in the bacteriomes and mycobiomes of sewage sludge (ss) and casts of earthworms *Dendrobaena hortensis* (Db), *Eisenia andrei* (Ea), *Eisenia fetida* (Ef), *Lumbricus friendi* (Lf), and *Lumbricus terrestris* (Lt). Supplementary Materials Figure S3: Alpha-diversity estimates of the bacteriomes and mycobiomes of sewage sludge (ss) and casts of earthworms *Dendrobaena hortensis* (Db), *Eisenia andrei* (Ea), *Eisenia fetida* (Ef), *Lumbricus friendi* (Lf), and *Lumbricus terrestris* (Lt). Asterisks denote statistically significant pairwise comparisons between ss and earthworm casts (** = Padj ≤ 0.01, *** = Padj ≤ 0.001). Supplementary Materials Figure S4: Beta-diversity estimates of the bacteriomes of sewage sludge (ss) and casts of earthworms *Dendrobaena hortensis* (Db), *Eisenia andrei* (Ea), *Eisenia fetida* (Ef), *Lumbricus friendi* (Lf), and *Lumbricus terrestris* (Lt). Supplementary Materials Figure S5: Abundance of selected functions of the bacteriomes of sewage sludge (ss) and casts of earthworms *Dendrobaena hortensis* (Db), *Eisenia andrei* (Ea), *Eisenia fetida* (Ef), *Lumbricus friendi* (Lf), and *Lumbricus terrestris* (Lt). Supplementary Materials Figure S6: Abundance of selected functions of the mycobiomes of sewage sludge (ss) and casts of earthworms *Dendrobaena hortensis* (Db), *Eisenia andrei* (Ea), *Eisenia fetida* (Ef), *Lumbricus friendi* (Lf), and *Lumbricus terrestris* (Lt). Tables: Supplementary Materials Table S1: Relative mean abundances of phyla and genera in the bacteriomes and mycobiomes of sewage sludge (ss) and casts of earthworms *Dendrobaena hortensis* (Db), *Eisenia andrei* (Ea), *Eisenia fetida* (Ef), *Lumbricus friendi* (Lf), and *Lumbricus terrestris* (Lt). Supplementary Materials Table S2: DESeq2 statistical tests of differential relative mean abundance of phyla and genera in the bacteriomes and mycobiomes of sewage sludge (ss) and casts of earthworms *Dendrobaena hortensis* (Db), *Eisenia andrei* (Ea), *Eisenia fetida* (Ef), *Lumbricus friendi* (Lf), and *Lumbricus terrestris* (Lt). Supplementary Materials Table S3: Alpha-diversity estimates of the bacteriomes and mycobiomes of sewage sludge (ss) and casts of earthworms *Dendrobaena hortensis* (Db), *Eisenia andrei* (Ea), *Eisenia fetida* (Ef), *Lumbricus friendi* (Lf), and *Lumbricus terrestris* (Lt). Supplementary Materials Table S4: Wilcoxon statistical tests of alpha-diversity estimates of the bacteriomes and mycobiomes of sewage sludge (ss) and casts of earthworms *Dendrobaena hortensis* (Db), *Eisenia andrei* (Ea), *Eisenia fetida* (Ef), *Lumbricus friendi* (Lf), and *Lumbricus terrestris* (Lt). Supplementary Materials Table S5: PERMANOVA tests of beta-diversity estimates of the bacteriomes and mycobiomes of sewage sludge (ss) and casts of earthworms *Dendrobaena hortensis* (Db), *Eisenia andrei* (Ea), *Eisenia fetida* (Ef), *Lumbricus friendi* (Lf), and *Lumbricus terrestris* (Lt). Supplementary Materials Table S6: Human bacterial pathogens and human fungal pathogens, and DESeq2 statistical tests of their differential relative mean abundance in the bacteriomes and mycobiomes of sewage sludge (ss) and casts of earthworms *Dendrobaena hortensis* (Db), *Eisenia andrei* (Ea), *Eisenia fetida* (Ef), *Lumbricus friendi* (Lf), and *Lumbricus terrestris* (Lt). Supplementary Materials Table S7: PERMANOVA tests of beta-diversity estimates in the functional bacteriomes of sewage sludge (ss) and casts of earthworms *Dendrobaena hortensis* (Db), *Eisenia andrei* (Ea), *Eisenia fetida* (Ef), *Lumbricus friendi* (Lf), and *Lumbricus terrestris* (Lt). Supplementary Materials Table S8: Wilcoxon statistical tests of selected functions of the bacteriomes of sewage sludge (ss) and casts of earthworms *Dendrobaena hortensis* (Db), *Eisenia andrei* (Ea), *Eisenia fetida* (Ef), *Lumbricus friendi* (Lf), and *Lumbricus terrestris* (Lt). Supplementary Materials Table S9: Wilcoxon statistical tests of selected functions of the mycobiomes of sewage sludge (ss) and casts of earthworms *Dendrobaena hortensis* (Db), *Eisenia andrei* (Ea), *Eisenia fetida* (Ef), *Lumbricus friendi* (Lf), and *Lumbricus terrestris* (Lt).

## References

[1] Tooraj M., Hooshyar H., Kimya P., Sheida A., Maryam S., Dariush M., Borhan A. (2023). Future of Sludge Management. Wastewater Treatment and Sludge Management Systems-The Gutter-to-Good Approaches.

[2] Eurostat Sewage Sludge Production and Disposal. https://ec.europa.eu/eurostat/databrowser/view/env_ww_spd/default/table?lang=en.

[3] Rorat A., Courtois P., Vandenbulcke F., Lemiere S. (2019). Sanitary and Environmental Aspects of Sewage Sludge Management. Industrial and Municipal Sludge.

[4] Shi Z., Xing K., Rameezdeen R., Chow C.W.K. (2024). Current Trends and Future Directions of Global Research on Wastewater to Energy: A Bibliometric Analysis and Review. Environ. Sci. Pollut. Res..

[5] Leino O., Äystö L., Fjäder P., Perkola N., Lehtoranta S. (2025). Contaminants of Environmental Concern in Sewage Sludge in the Nordic Countries. Environ. Pollut..

[6] Domínguez J., Ray S. (2018). Earthworms and Vermicomposting. Earthworms—The Ecological Engineers of Soil.

[7] Domínguez J., Edwards C.A. (2004). State of the Art and New Perspectives on Vermicomposting Research. Earthworm Ecology.

[8] Georgi K., Ekaterina S., Alexander P., Alexander R., Kirill Y., Andrey V. (2022). Sewage Sludge as an Object of Vermicomposting. Bioresour. Technol. Rep..

[9] Dominguez J., Aira M., Crandall K.A., Perez-Losada M. (2021). Earthworms Drastically Change Fungal and Bacterial Communities during Vermicomposting of Sewage Sludge. Sci. Rep..

[10] Dominguez J., Aira M., Kolbe A.R., Gomez-Brandon M., Perez-Losada M. (2019). Changes in the Composition and Function of Bacterial Communities during Vermicomposting May Explain Beneficial Properties of Vermicompost. Sci. Rep..

[11] Zhao C., Wang Y., Wang Y., Wu F., Zhang J., Cui R., Wang L., Mu H. (2018). Insights into the Role of Earthworms on the Optimization of Microbial Community Structure during Vermicomposting of Sewage Sludge by PLFA Analysis. Waste Manag..

[12] Gómez-Roel A., Aira M., Domínguez J. (2024). Vermicomposting Enhances Microbial Detoxification of Sewage Sludge, Enabling Potential Application of the Treated Product in Agroecosystems. Appl. Sci..

[13] Hrčka M., Hřebečková T., Hanč A., Grasserová A., Cajthaml T. (2024). Changes in the Content of Emerging Pollutants and Potentially Hazardous Substances during Vermi/Composting of a Mixture of Sewage Sludge and Moulded Pulp. Environ. Pollut..

[14] Aira M., Pérez-Losada M., Crandall K.A., Domínguez J. (2022). Host Taxonomy Determines the Composition, Structure, and Diversity of the Earthworm Cast Microbiome under Homogenous Feeding Conditions. FEMS Microbiol. Ecol..

[15] Suthar S. (2008). Microbial and Decomposition Efficiencies of Monoculture and Polyculture Vermireactors, Based on Epigeic and Anecic Earthworms. World J. Microbiol. Biotechnol..

[16] Edwards C.A., Bohlen P.J. (1996). Biology and Ecology of Earthworms.

[17] Arnold R.E., Hodson M.E. (2007). Effect of Time and Mode of Depuration on Tissue Copper Concentrations of the Earthworms *Eisenia andrei*, *Lumbricus rubellus* and *Lumbricus terrestris*. Environ. Pollut..

[18] Hartenstein F., Hartenstein E., Hartenstein R. (1981). Gut Load and Transit Time in the Earthworm *Eisenia foetida*. Pedobiologia.

[19] Dash H.K., Beuka B.N., Dash M.C. (1986). Gut Load, Transit Time, Gut Microflora and Turnover of Soil, Plant and Fungal Material by Some Tropical Earthworms. Pedobiologia.

[20] Callahan B.J., McMurdie P.J., Rosen M.J., Han A.W., Johnson A.J., Holmes S.P. (2016). DADA2: High-Resolution Sample Inference from Illumina Amplicon Data. Nat. Methods.

[21] Callahan B.J., McMurdie P.J., Holmes S.P. (2017). Exact Sequence Variants Should Replace Operational Taxonomic Units in Marker-Gene Data Analysis. ISME J..

[22] Wang Q., Garrity G.M., Tiedje J.M., Cole J.R. (2007). Naive Bayesian Classifier for Rapid Assignment of rRNA Sequences into the New Bacterial Taxonomy. Appl. Environ. Microb..

[23] Quast C., Pruesse E., Yilmaz P., Gerken J., Schweer T., Yarza P., Peplies J., Glockner F.O. (2013). The SILVA Ribosomal RNA Gene Database Project: Improved Data Processing and Web-Based Tools. Nucleic Acids Res..

[24] Nilsson R.H., Larsson K.H., Taylor A.F.S., Bengtsson-Palme J., Jeppesen T.S., Schigel D., Kennedy P., Picard K., Glockner F.O., Tedersoo L. (2019). The UNITE Database for Molecular Identification of Fungi: Handling Dark Taxa and Parallel Taxonomic Classifications. Nucleic Acids Res..

[25] Love M.I., Huber W., Anders S. (2014). Moderated Estimation of Fold Change and Dispersion for RNA-Seq Data with DESeq2. Genome Biol..

[26] Aira M., Domínguez J. (2025). Effect of Earthworm Digestion on Abundance, Composition and Diversity of Bacterial Pathogens in Sewage Sludge from Wastewater Treatment Plants. Microorganisms.

[27] Edgar R.C. (2018). Updating the 97% Identity Threshold for 16S Ribosomal RNA OTUs. Bioinformatics.

[28] Bartlett A., Padfield D., Lear L., Bendall R., Vos M. (2022). A Comprehensive List of Bacterial Pathogens Infecting Humans. Microbiology.

[29] Aira M., Gómez-Roel A., Domínguez J. (2025). Earthworms Significantly Alter the Composition, Diversity, Abundance and Pathogen Load of Fungal Communities in Sewage Sludge from Different Urban Wastewater Treatment Plants. Pathogens.

[30] Nguyen N.H., Song Z., Bates S.T., Branco S., Tedersoo L., Menke J., Schilling J.S., Kennedy P.G. (2016). FUNGuild: An Open Annotation Tool for Parsing Fungal Community Datasets by Ecological Guild. Fungal. Ecol..

[31] Alvarez-Jarreta J., Amos B., Aurrecoechea C., Bah S., Barba M., Barreto A., Basenko E.Y., Belnap R., Blevins A., Böhme U. (2024). VEuPathDB: The Eukaryotic Pathogen, Vector and Host Bioinformatics Resource Center in 2023. Nucleic Acids Res..

[32] World Health Organization (2022). WHO Fungal Priority Pathogens List to Guide Research, Development and Public Health Action.

[33] American Society for Microbiology (2019). One Health: Fungal Pathogens of Humans, Animals, and Plants: Report on an American Academy of Microbiology Colloquium Held in Washington, DC, on October 18, 2017.

[34] Assress H.A., Selvarajan R., Nyoni H., Ntushelo K., Mamba B.B., Msagati T.A.M. (2019). Diversity, Co-Occurrence and Implications of Fungal Communities in Wastewater Treatment Plants. Sci. Rep..

[35] Douglas G.M., Maffei V.J., Zaneveld J.R., Yurgel S.N., Brown J.R., Taylor C.M., Huttenhower C., Langille M.G.I. (2020). PICRUSt2 for Prediction of Metagenome Functions. Nat. Biotechnol..

[36] Tenenbaum D. (2016). KEGGREST: Client-Side REST Access to KEGG.

[37] McMurdie P.J., Holmes S. (2013). Phyloseq: An R Package for Reproducible Interactive Analysis and Graphics of Microbiome Census Data. PLoS ONE.

[38] Wickham H., Averick M., Bryan J., Chang W., McGowan L.D., François R., Grolemund G., Hayes A., Henry L., Hester J. (2019). Welcome to the Tidyverse. J. Open Source Softw..

[39] Oksanen J., Simpson G.L., Blanchet F.G., Kindt R., Legendre P., Minchin P.R., O’Hara R.B., Solymos P., Stevens M.H.H., Szoecs E. (2026). Vegan: Community Ecology Package.

[40] Kassambara A. (2025). Rstatix: Pipe-Friendly Framework for Basic Statistical Tests.

[41] Liu C., Cui Y., Li X., Yao M. (2021). Microeco: An R Package for Data Mining in Microbial Community Ecology. FEMS Microbiol. Ecol..

[42] Bhatti A.A., Haq S., Bhat R.A. (2017). Actinomycetes Benefaction Role in Soil and Plant Health. Microb. Pathog..

[43] Barka E.A., Vatsa P., Sanchez L., Gaveau-Vaillant N., Jacquard C., Klenk H.-P., Clément C., Ouhdouch Y., van Wezel G.P. (2015). Taxonomy, Physiology, and Natural Products of Actinobacteria. Microbiol. Mol. Biol. Rev..

[44] López-González J.A., Estrella-González M.J., Lerma-Moliz R., Jurado M.M., Suárez-Estrella F., López M.J. (2021). Industrial Composting of Sewage Sludge: Study of the Bacteriome, Sanitation, and Antibiotic-Resistant Strains. Front. Microbiol..

[45] Xia H., Chen J., Chen X., Huang K., Wu Y. (2019). Effects of Tetracycline Residuals on Humification, Microbial Profile and Antibiotic Resistance Genes during Vermicomposting of Dewatered Sludge. Environ. Pollut..

[46] Hao J., Wang L., Aspe N.M., Han A.C., Chen M., Li M., Zhang S., Wu D. (2024). Seasonal Dynamics of Gut Microbiome: A Study of Multi-Kingdom Microbiota of Earthworm Gut in an Urban Park. Appl. Soil Ecol..

[47] Sapkota R., Santos S., Farias P., Krogh P.H., Winding A. (2020). Insights into the Earthworm Gut Multi-Kingdom Microbial Communities. Sci. Total Environ..

[48] Thakur S.S., Lone A.R., Tiwari N., Jain S.K., Yadav S. (2021). Metagenomic Exploration of Bacterial Community Structure of Earthworms’ Gut. J. Pure Appl. Microbiol..

[49] Liu N., Graham D.W., Zhao Y., Yang X.-R., Li G., Zhu Y.-G. (2025). Role of Earthworms and Their Excretion Products in Reducing Antimicrobial Resistance and Putative Pathogens during Vermicomposting. Chem. Eng. J..

[50] Eastman B.R., Kane P.N., Edwards C.A., Trytek L., Gunadi B., Stermer A.L., Mobley J.R. (2001). The Effectiveness of Vermiculture in Human Pathogen Reduction for USEPA Biosolids Stabilization. Compost. Sci. Util..

[51] Grantina-Ievina L., Rodze I., Meghvansi M.K., Varma A. (2020). Survival of Pathogenic and Antibiotic-Resistant Bacteria in Vermicompost, Sewage Sludge, and Other Types of Composts in Temperate Climate Conditions. Biology of Composts.

[52] Wang N., Wang W., Jiang Y., Dai W., Li P., Yao D., Wang J., Shi Y., Cui Z., Cao H. (2021). Variations in Bacterial Taxonomic Profiles and Potential Functions in Response to the Gut Transit of Earthworms (*Eisenia fetida*) Feeding on Cow Manure. Sci. Total Environ..

[53] Aira M., Domínguez J. (2014). Changes in Nutrient Pools, Microbial Biomass and Microbial Activity in Soils after Transit through the Gut of Three Endogeic Earthworm Species of the Genus Postandrilus Qui and Bouché, 1998. J. Soils Sediments.

[54] De Simeis D., Serra S. (2021). Actinomycetes: A Never-Ending Source of Bioactive Compounds—An Overview on Antibiotics Production. Antibiotics.

[55] Arora S., Saraswat S., Rajpal A., Shringi H., Mishra R., Sethi J., Rajvanshi J., Nag A., Saxena S., Kazmi A.A. (2021). Effect of Earthworms in Reduction and Fate of Antibiotic Resistant Bacteria (ARB) and Antibiotic Resistant Genes (ARGs) during Clinical Laboratory Wastewater Treatment by Vermifiltration. Sci. Total Environ..

[56] Huang K., Xia H., Wu Y., Chen J., Cui G., Li F., Chen Y., Wu N. (2018). Effects of Earthworms on the Fate of Tetracycline and Fluoroquinolone Resistance Genes of Sewage Sludge during Vermicomposting. Bioresour. Technol..

[57] Louca S., Polz M.F., Mazel F., Albright M.B.N., Huber J.A., O’Connor M.I., Ackermann M., Hahn A.S., Srivastava D.S., Crowe S.A. (2018). Function and Functional Redundancy in Microbial Systems. Nat. Ecol. Evol..

