# Supplementary material for: Earthworms Drastically and Differentially Modify the Bacteriomes and Mycobiomes of Sewage Sludge
Source: BioTech (Basel). 2026 May 10;15(2):33. doi: 10.3390/biotech15020033 (PMC13214669; doi:10.3390/biotech15020033)
Supplement: Supplementary file 1 [file biotech-15-00033-s001.zip › Figure S2.pdf]

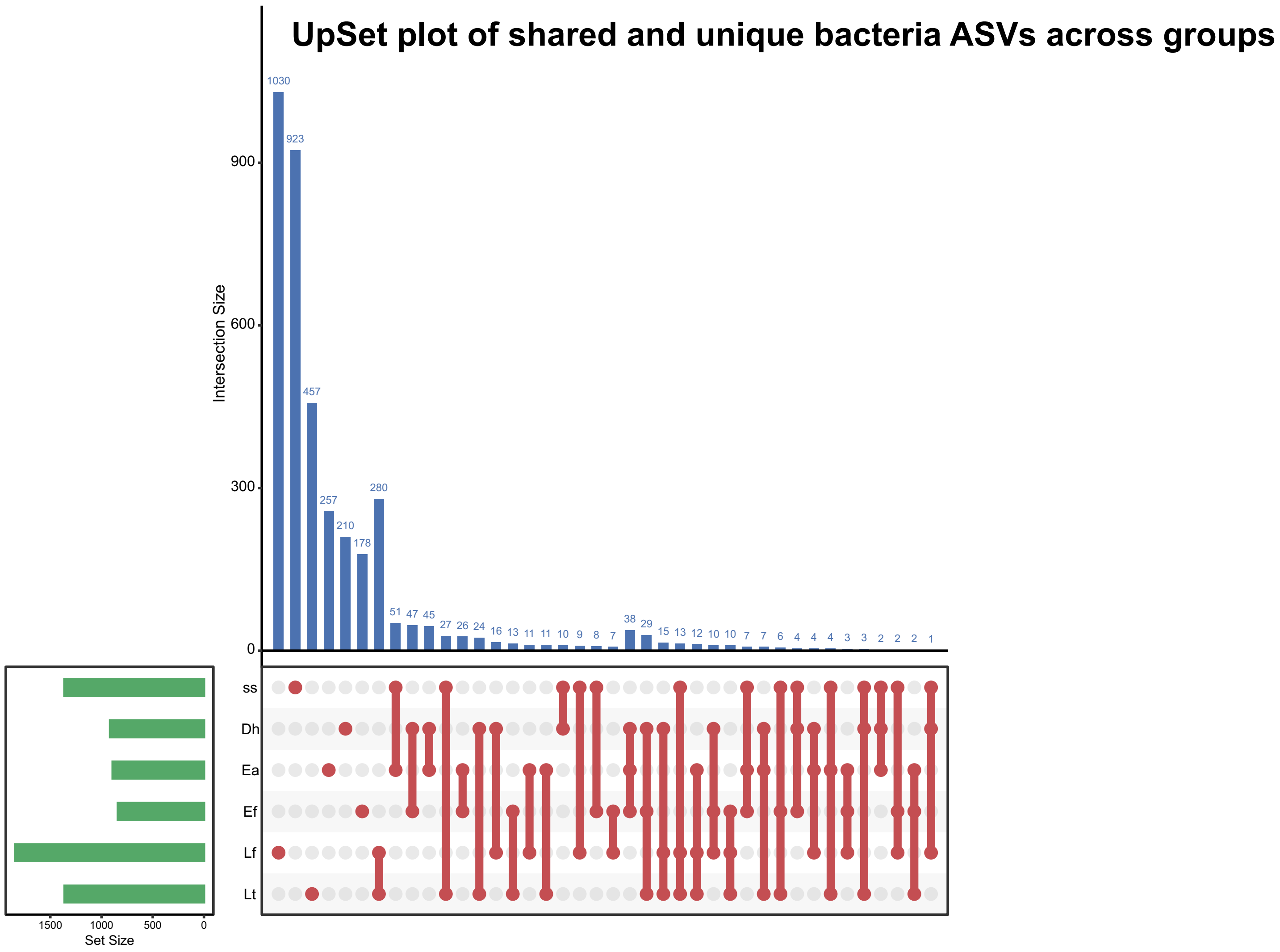
UpSet plot of shared and unique bacteria ASVs across groups
Intersection Size
900
600
300
0
1030 923 457 257 210 178 280 51 47 45 27 26 24 16 13 11 11 10 9 8 7 38 29 15 13 12 10 10 7 7 6 4 4 4 3 3 2 2 2 1
ss
Dh
Ea
Ef
Lf
Lt
1500
1000
500
0
Set Size

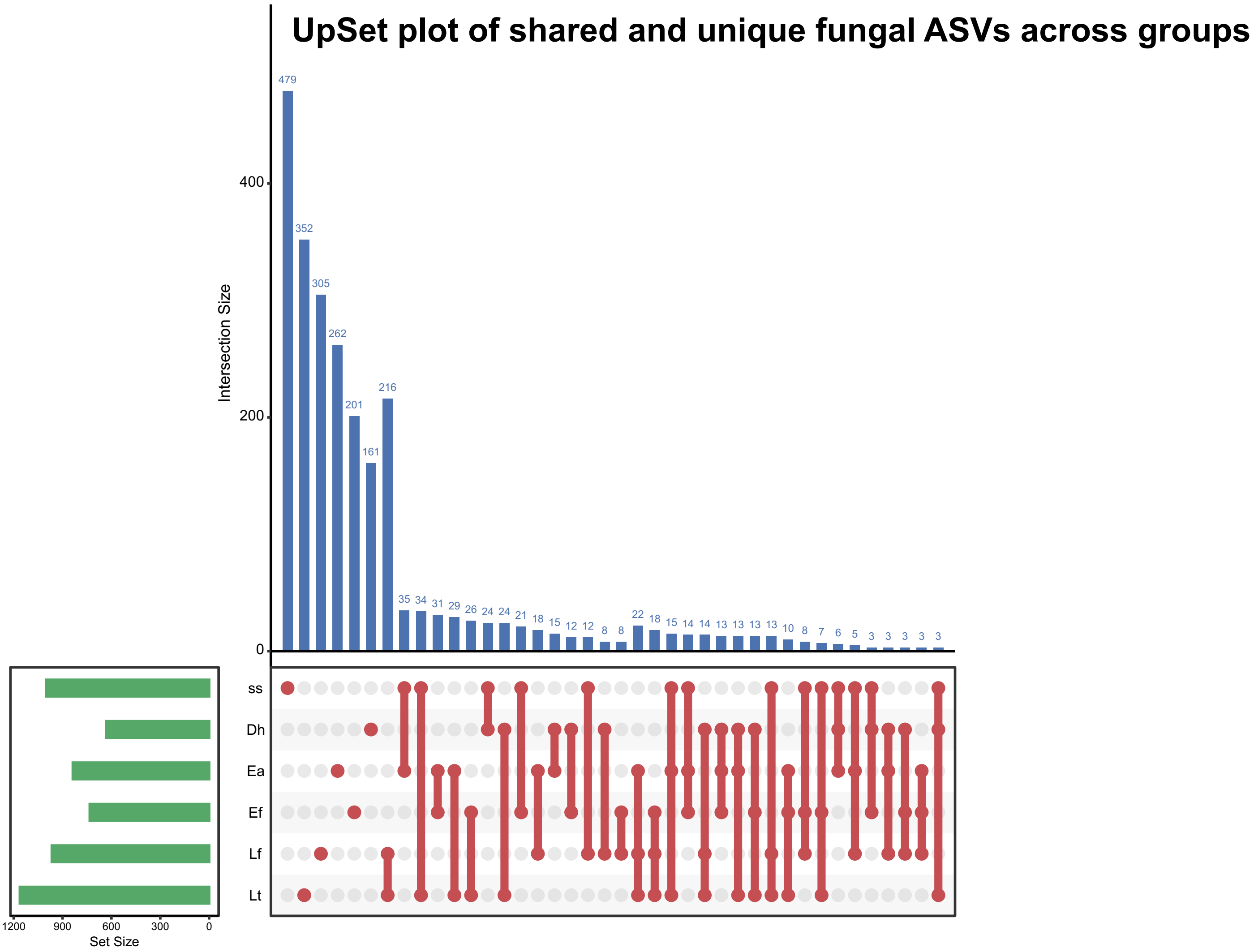
UpSet plot of shared and unique fungal ASVs across groups
Intersection Size
400
200
0
479 352 305 262 201 161 216 35 34 31 29 26 24 24 21 18 15 12 12 8 8 22 18 15 14 14 13 13 13 13 10 8 7 6 5 3 3 3 3 3
ss
Dh
Ea
Ef
Lf
Lt
1200
900
600
300
0
Set Size
