# Supplementary figures and images for: Earthworms Drastically and Differentially Modify the Bacteriomes and Mycobiomes of Sewage Sludge
Source: BioTech (Basel). 2026 May 10;15(2):33. doi: 10.3390/biotech15020033 (PMC13214669; doi:10.3390/biotech15020033)

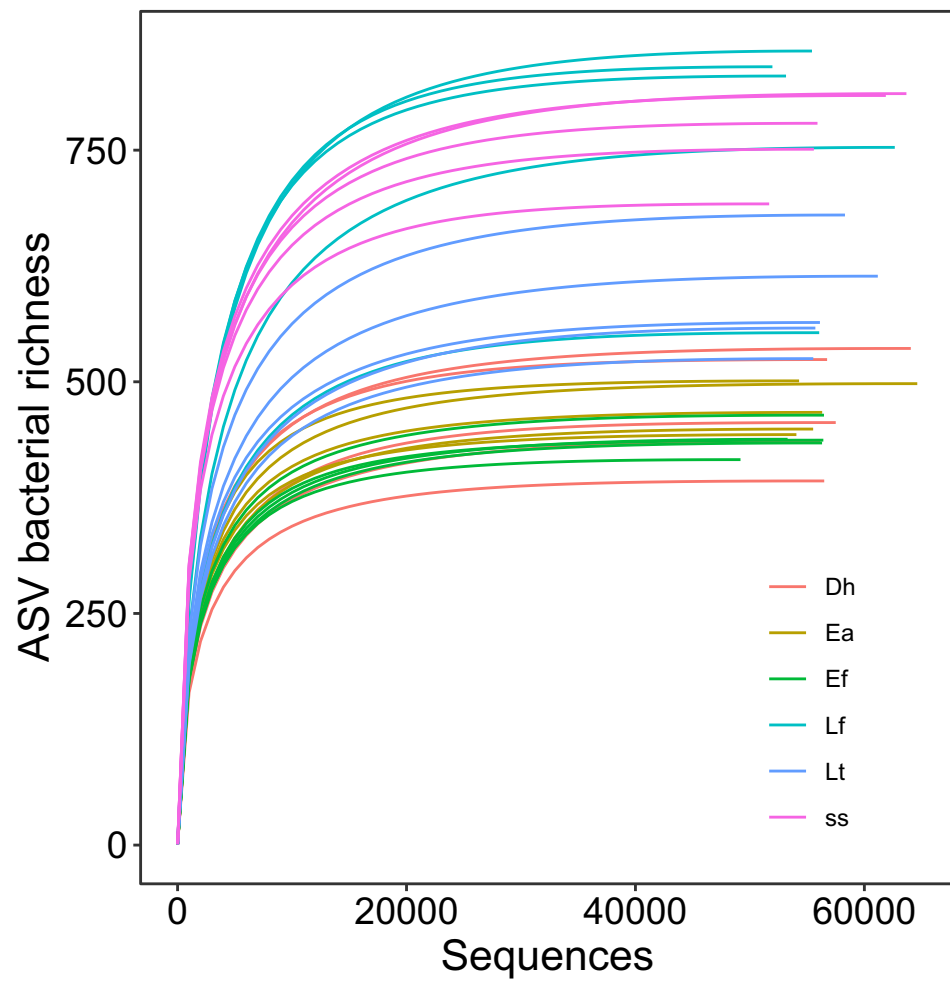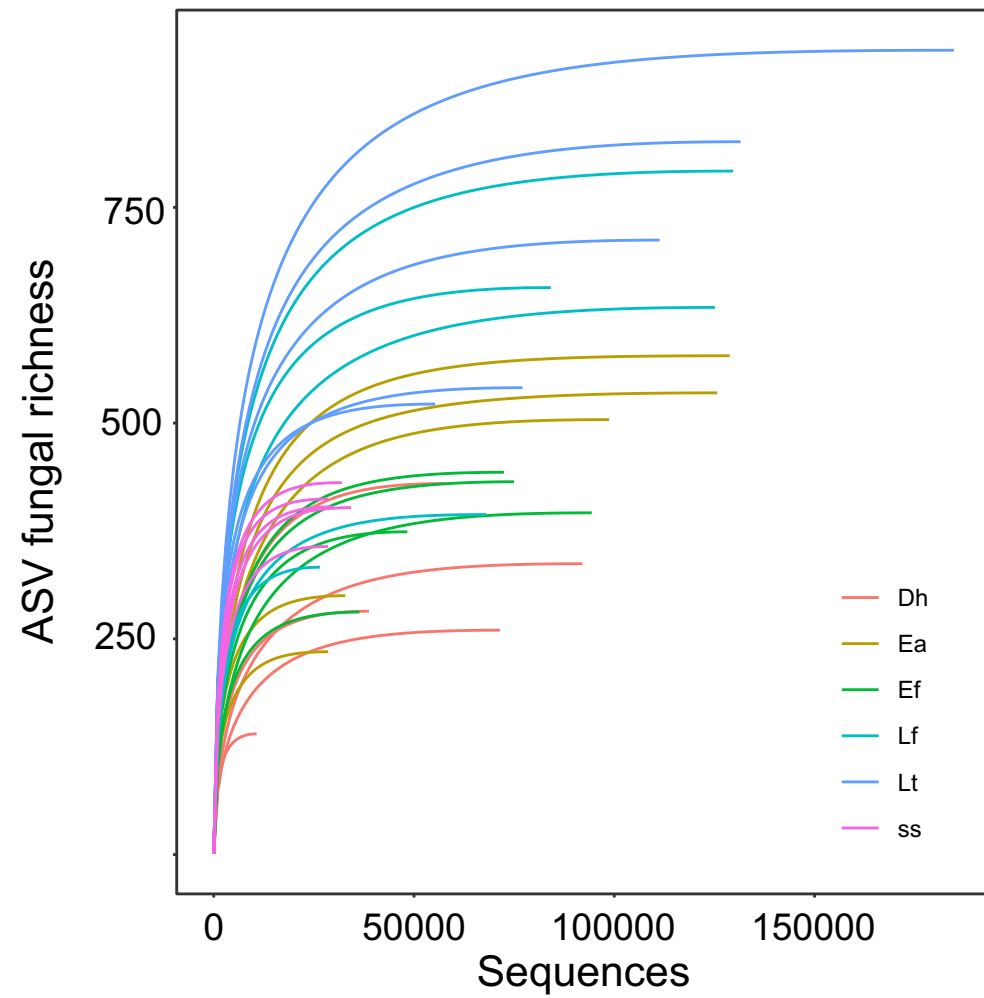

Supplement: Supplementary file 1 [file biotech-15-00033-s001.zip › Figure S1.pdf]

Bacterial diversity

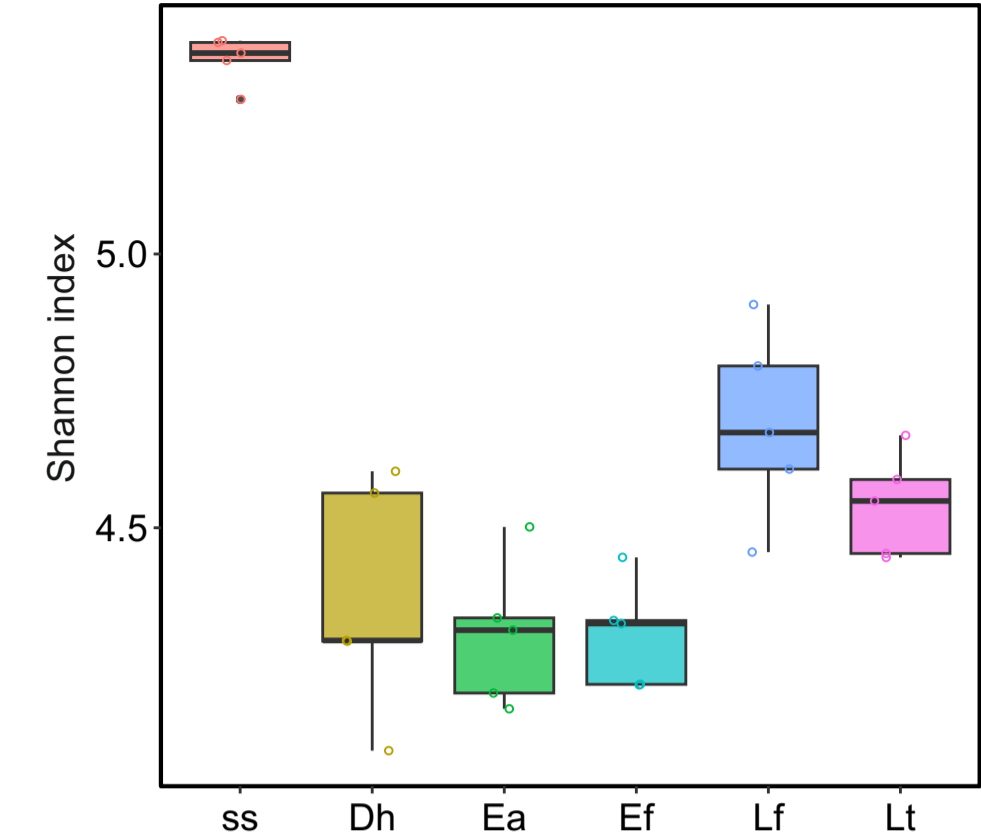

Bacterial phylogenetic diversity

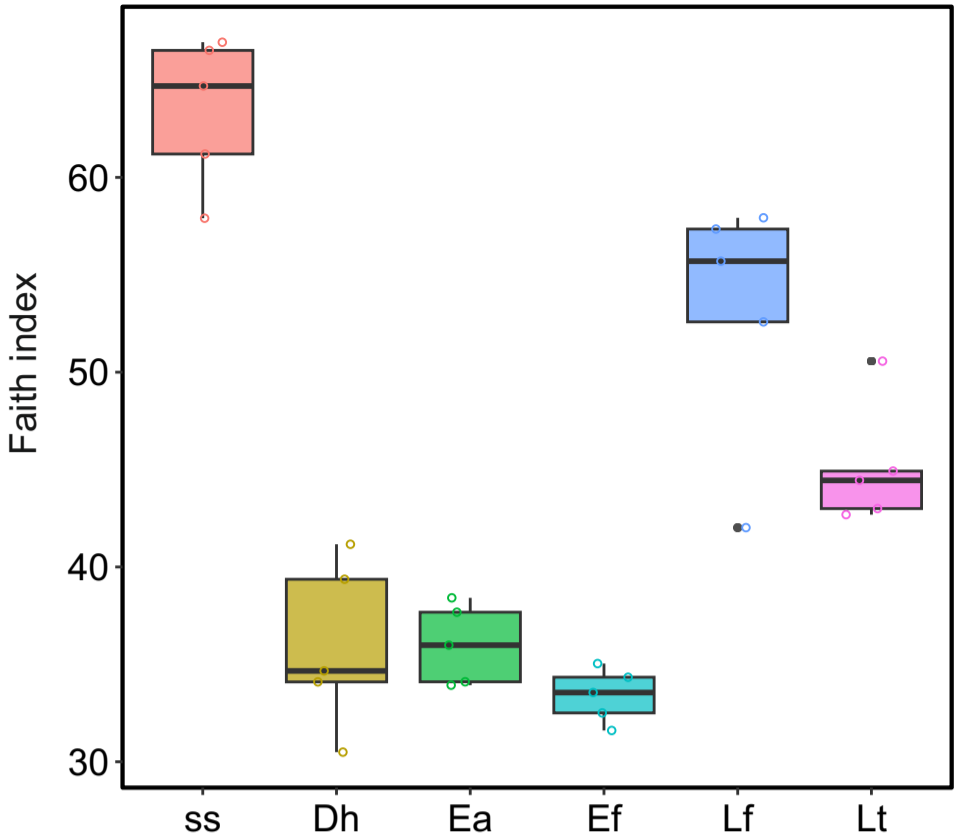

Fungal diversity

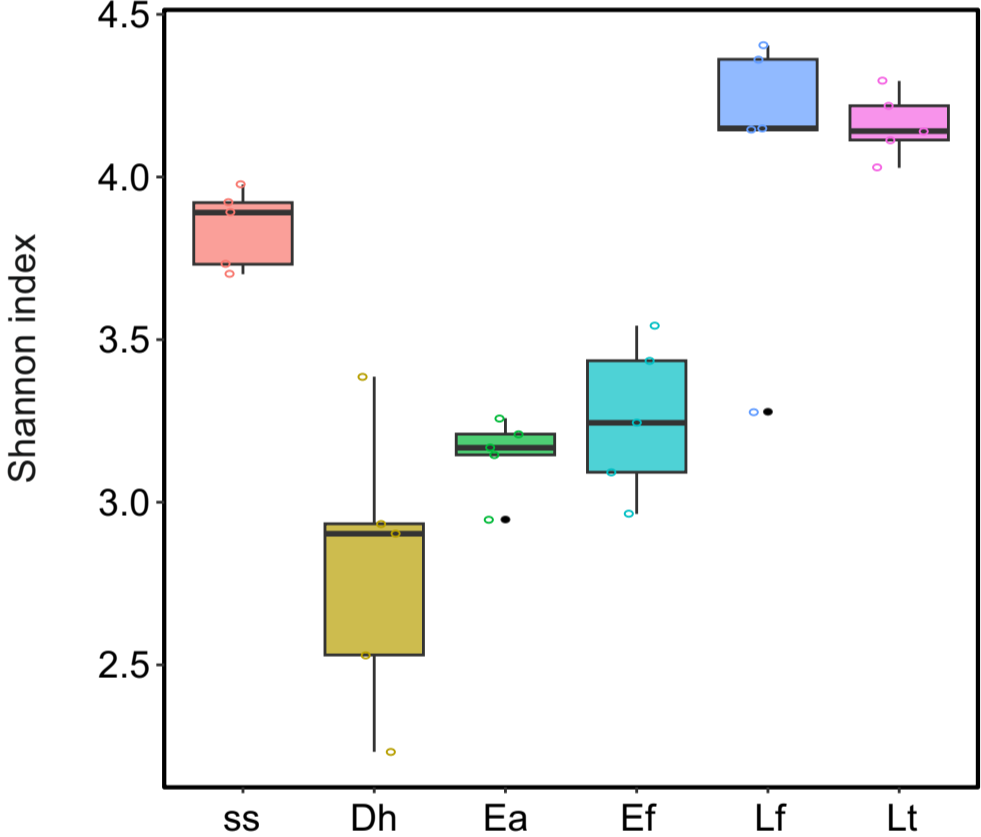

Supplement: Supplementary file 1 [file biotech-15-00033-s001.zip › Figure S3.pdf]

**PCoA Weigthed UNIFRAC**

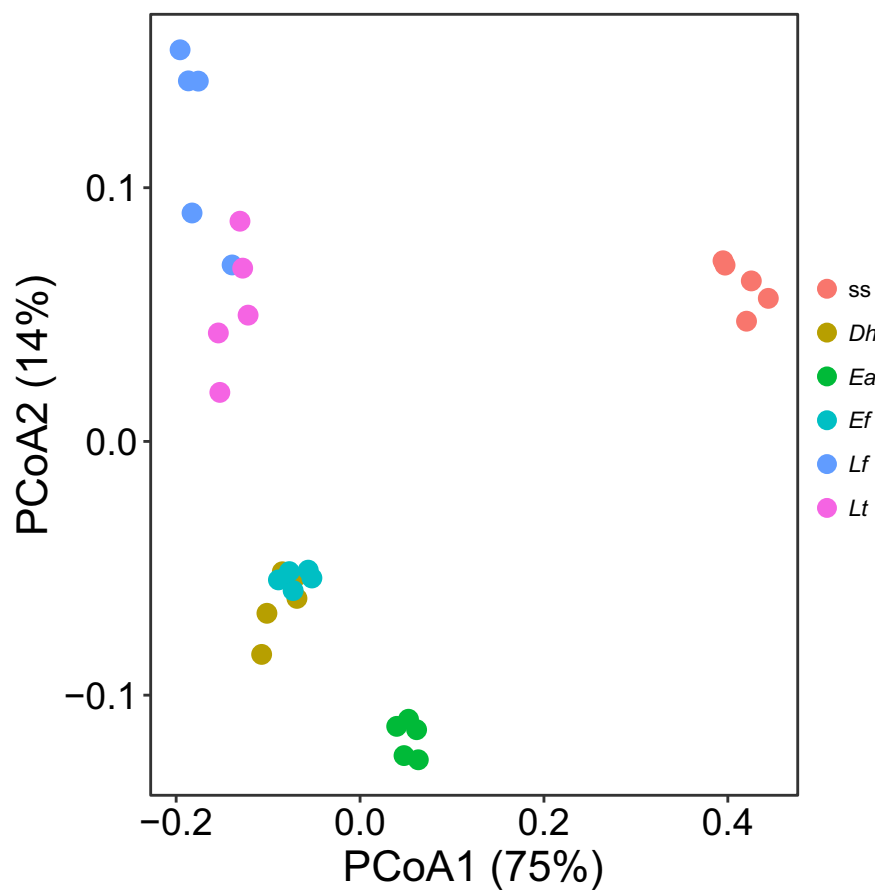

**PCoA Unweigthed UNIFRAC**

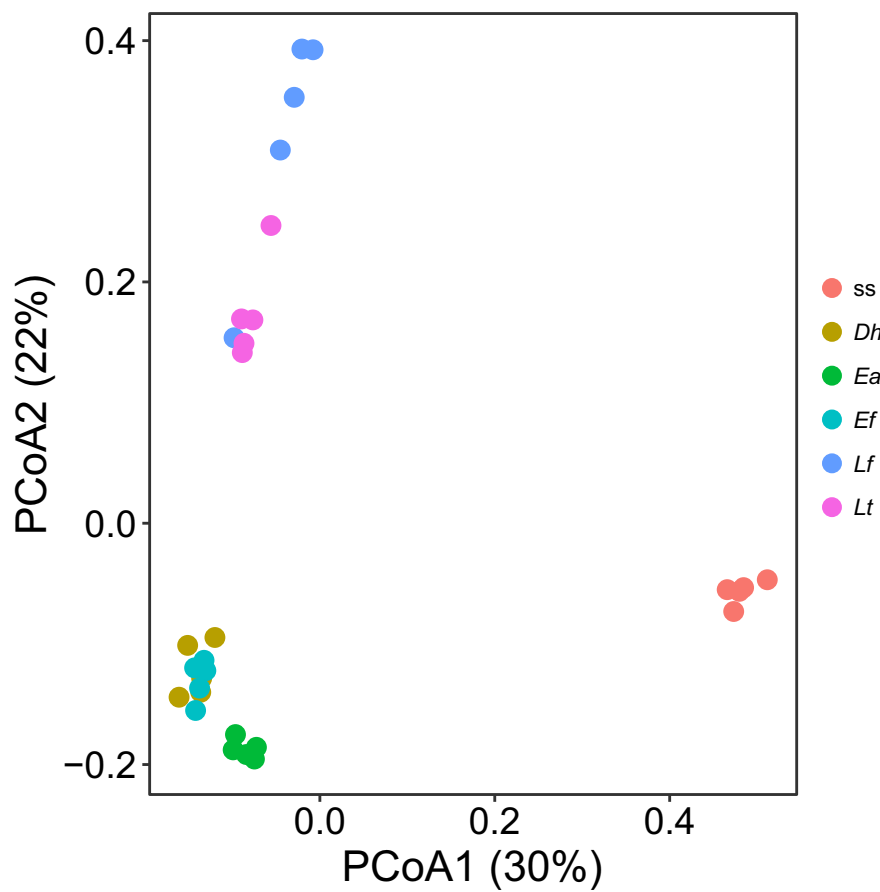

Supplement: Supplementary file 1 [file biotech-15-00033-s001.zip › Figure S4.pdf]

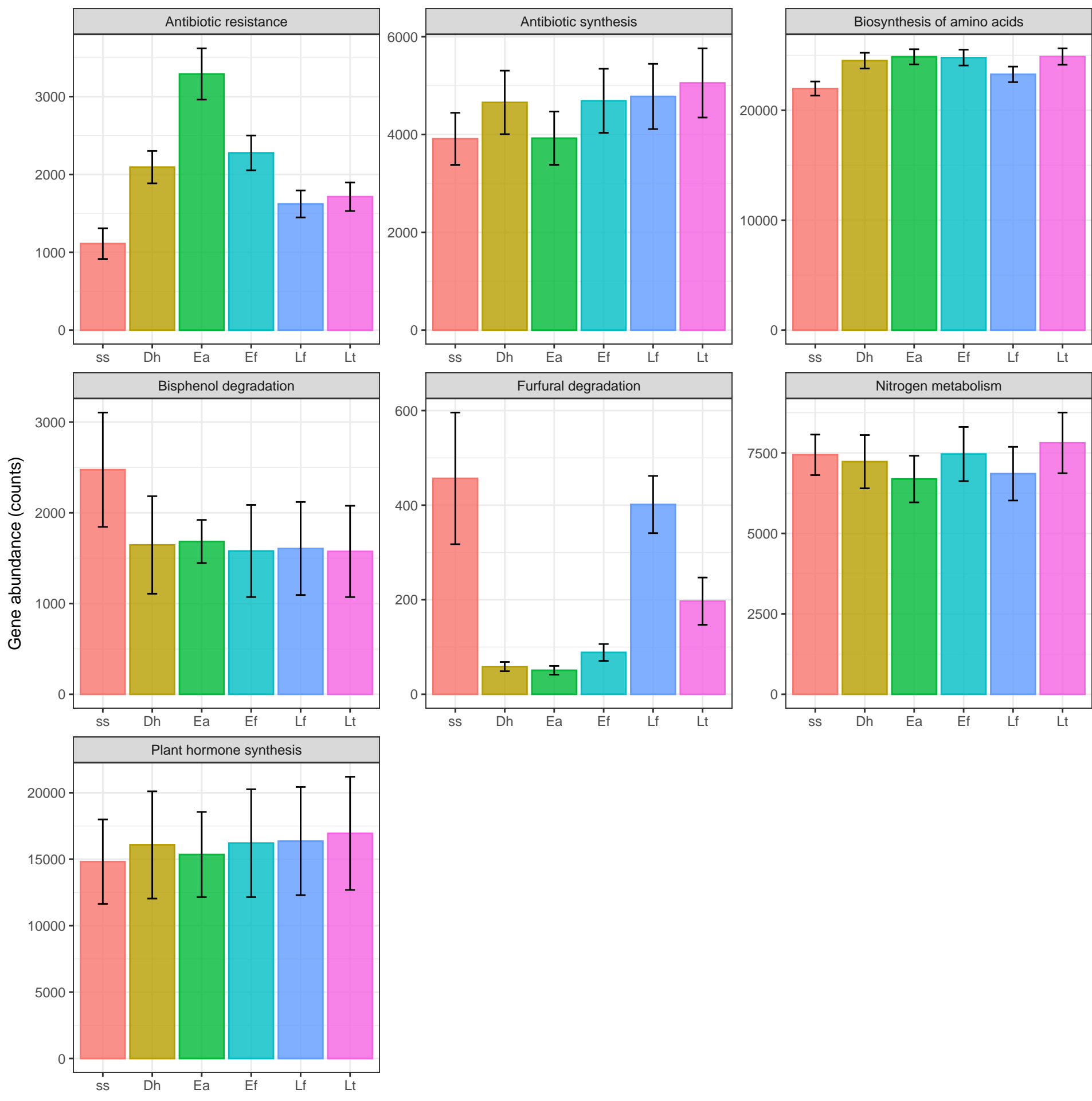

Supplement: Supplementary file 1 [file biotech-15-00033-s001.zip › Figure S5.pdf]

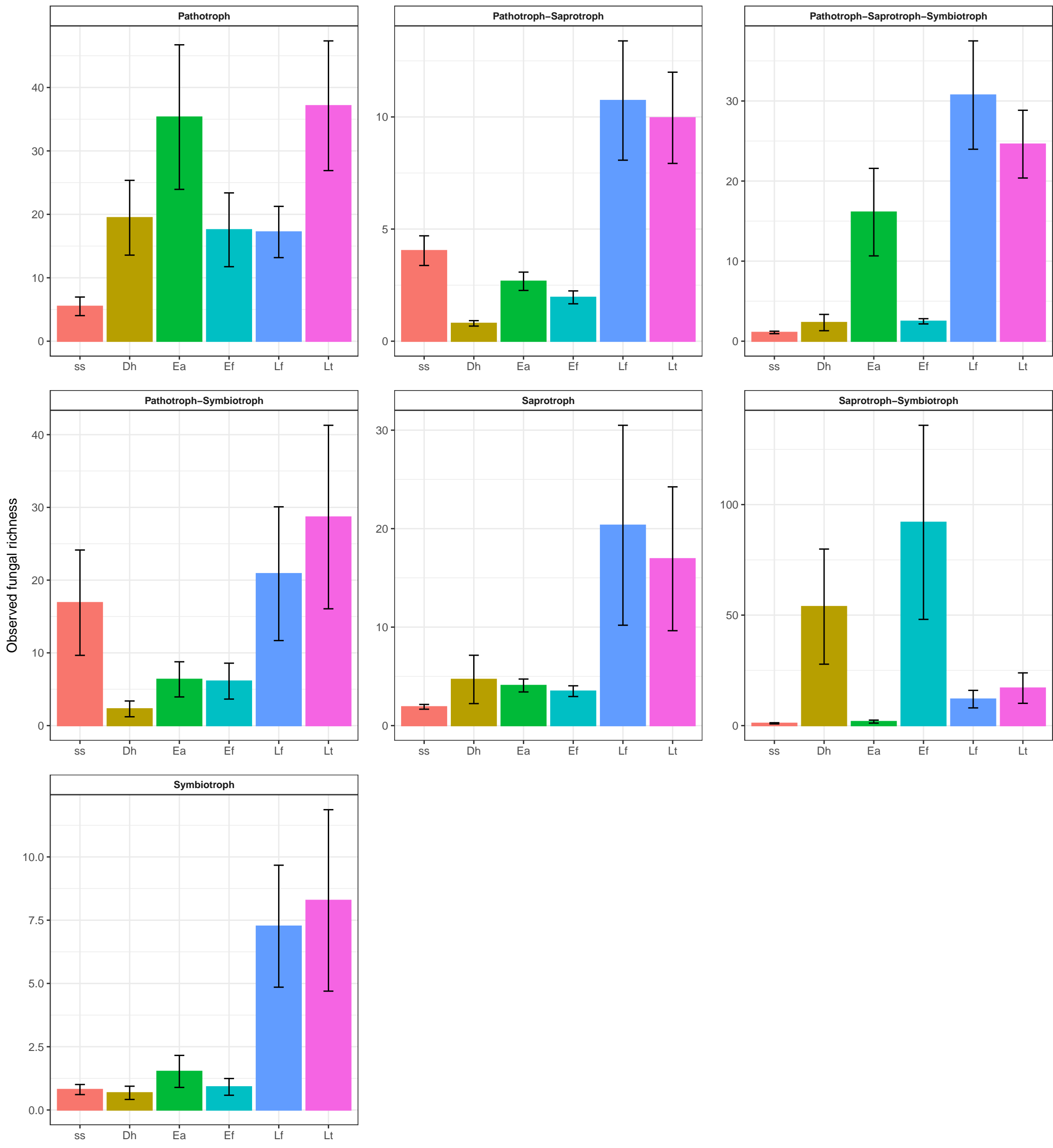

Supplement: Supplementary file 1 [file biotech-15-00033-s001.zip › Figure S6.pdf]
